# When does speech sound disorder matter for literacy? The role of disordered speech errors, co‐occurring language impairment and family risk of dyslexia

**DOI:** 10.1111/jcpp.12648

**Published:** 2016-11-07

**Authors:** Marianna E. Hayiou‐Thomas, Julia M. Carroll, Ruth Leavett, Charles Hulme, Margaret J. Snowling

**Affiliations:** ^1^ Department of PsychologyUniversity of YorkYorkUK; ^2^ Centre for Research in Psychology, Behaviour and AchievementCoventry UniversityCoventryUK; ^3^ Children's Therapy ServiceYork Teaching Hospital NHS Foundation TrustYorkUK; ^4^ Division of Psychology and Language SciencesUniversity College LondonLondonUK; ^5^ Department of Experimental Psychology and St John's CollegeUniversity of OxfordOxfordUK

**Keywords:** Speech sound disorder, literacy, language impairment, disordered speech errors, family risk of dyslexia

## Abstract

**Background:**

This study considers the role of early speech difficulties in literacy development, in the context of additional risk factors.

**Method:**

Children were identified with speech sound disorder (SSD) at the age of 3½ years, on the basis of performance on the Diagnostic Evaluation of Articulation and Phonology. Their literacy skills were assessed at the start of formal reading instruction (age 5½), using measures of phoneme awareness, word‐level reading and spelling; and 3 years later (age 8), using measures of word‐level reading, spelling and reading comprehension.

**Results:**

The presence of early SSD conferred a small but significant risk of poor phonemic skills and spelling at the age of 5½ and of poor word reading at the age of 8. Furthermore, within the group with SSD, the persistence of speech difficulties to the point of school entry was associated with poorer emergent literacy skills, and children with ‘disordered’ speech errors had poorer word reading skills than children whose speech errors indicated ‘delay’. In contrast, the initial severity of SSD was not a significant predictor of reading development. Beyond the domain of speech, the presence of a co‐occurring language impairment was strongly predictive of literacy skills and having a family risk of dyslexia predicted additional variance in literacy at both time‐points.

**Conclusions:**

Early SSD alone has only modest effects on literacy development but when additional risk factors are present, these can have serious negative consequences, consistent with the view that multiple risks accumulate to predict reading disorders.

## Introduction

Developmental speech sound disorder (SSD) encompasses a wide range of difficulties with the production of speech in children, and is defined in DSM‐5 (American Psychiatric Association, 2013) as a ‘persistent difficulty with speech sound production that interferes with speech intelligibility or prevents verbal communication’ that cannot be explained in terms of sensory problems, motoric difficulties or other physical conditions. Children with SSD make systematic omissions, substitutions or distortions of phonemes within words, despite being able to repeat these phonemes in isolation. The prevalence of SSD in 4‐ to 6‐year‐old children has been estimated at approximately 3–6%, based on large population‐based cohorts (Beitchman et al., 1986; Eadie et al., 2015; Shriberg, Tomblin, & McSweeny, 1999), although estimates vary substantially with age and with diagnostic criteria. Speech difficulties in young children often arouse their parents’ concern, and are a frequent cause of referral to speech and language therapy services (Bishop & Hayiou‐Thomas, 2008; Zhang & Tomblin, 2000). Although such difficulties often resolve (Dodd, 1995), they are commonly seen as a risk factor for literacy difficulties (Bird, Bishop, & Freeman, 1995).

A separate line of research suggests that speech and literacy difficulties share a common genetic aetiology (Hayiou‐Thomas, Harlaar, Dale, & Plomin, 2010; Lewis et al., 2011; Stein et al., 2004). The relationship between them is not, however, straightforward, rather the risk of poor literacy in SSD may be moderated by other factors including the persistence of speech difficulties to the age at which formal reading instruction begins; the nature of the speech errors made by the child; co‐occurring language difficulties; and a family risk of dyslexia (Pennington & Bishop, 2009). While previous research has examined each of these factors in isolation, they have not been examined in combination, which is crucial for understanding their relative importance.

The persistence of SSD into the early school years is thought to be problematic for literacy development, not least because the phonological skills which are affected include those which are a crucial foundation for learning to read. According to the ‘critical age hypothesis’, speech deficits matter if they are present at the time that a child is learning to read (Bishop & Adams, 1990). The strongest evidence in support of a role for SSD per se comes from a study by Bird et al. (1995), who followed a group of 5‐ to 7‐year‐old children with SSD and found that, regardless of the presence of additional language impairment, the presence of speech difficulties at school entry posed a significant barrier to learning to read.

A weaker role for SSD was reported by Nathan, Stackhouse, Goulandris, and Snowling (2004), who followed a sample of children with SSD from 4½ to 6½ years and found that, although the presence of SSD at 6½ was strongly related to poor phonological awareness and literacy skills, this was largely due to co‐occurring language difficulties. Nonetheless, SSD status at 6½ on its own accounted for a small but significant amount of unique variance in spelling but not reading skills. Similar findings, of weak emergent literacy skills in children with isolated SSD, were also reported by DeThorne et al. (2006), and there is evidence of long‐term effects of early isolated SSD when literacy skills were measured in middle childhood (Lewis, Freebairn, & Taylor, 2000).

An alternative view focuses not on the timing of SSD, but rather on the characteristics of the speech errors made. Dodd (1995) classified children according to whether they showed either developmental delay (speech errors that are prevalent in younger, typically developing children) or disordered speech errors (errors that do not tend to occur in typically developing speech, such as vowel distortions or initial consonant deletions). According to Dodd, children showing disordered speech errors are likely to have deficits in the way that they represent the phonology of known words, and are at high risk of reading problems because phonological awareness skills are compromised. On the other hand, it is assumed that children with speech delay will at some point catch up to their typical peers.

Evidence supporting this view comes from comparisons of children with speech delay and speech disorder on phonological processing measures. Holm, Farrier, and Dodd (2008) found that, although the groups were similar in terms of language skills, the children who made disordered speech errors showed difficulties on phonological awareness tasks in comparison to those with speech delay, indicating difficulties in learning the constraints of a phonological system. Similarly, Leitão, Hogben, and Fletcher (1997) found that only children with disordered speech errors had difficulties with phonological awareness. A follow‐up at 12 years old (Leitao & Fletcher, 2004) showed that most of the sample continued to have literacy difficulties, and that the children with disordered speech at school entry were particularly impaired. These longitudinal findings were replicated in an independent sample of preschoolers with SSD: children who made disordered speech sound errors were more likely to have weak literacy skills at the age of 8 (Preston, Hull, & Edwards, 2013).

Beyond the nature and timing of the SSD itself, broader language difficulties have a substantial impact on literacy development. The most direct attempt to disentangle whether it is the persistence of SSD or its co‐occurrence with LI that matters for literacy, was a study by Raitano, Pennington, Tunick, Boada, and Shriberg (2004). Kindergarten children (age 5–6 years) who had a history of SSD were categorised according to their current SSD status (resolved or persistent) and LI status. Both current SSD and LI had significant, additive, effects on phonological awareness skills; additionally, a history of SSD, even if it had resolved, had a significant effect on phonological awareness at age 6. When these children were 7–9 years, Peterson, Pennington, and Shriberg (2009) reported that while both co‐occurring LI and persistent SSD continued to predict variance in phonological awareness skills, only LI predicted reading outcomes. That is, although children with a history of SSD had phonological processing deficits well into middle childhood, this only appeared to result in a reading deficit if they also had broader oral language difficulties.

In summary, the relationship between SSD, LI and Reading Disorder is complex: they co‐occur more often than would be expected by chance, probably because of overlapping genetic and environmental aetiology (Pennington & Bishop, 2009). A reasonable interpretation of current findings would be that, as both phonological and broader language skills are a foundation for reading (Hulme, Nash, Gooch, Lervåg, & Snowling, 2015) deficits in these skills are endophenotypes that mediate the well‐established genetic risk for dyslexia on reading development (Lewis et al., 2011). Importantly however, Carroll, Mundy, and Cunningham (2014) reported that a family history of dyslexia conferred additional risk of poor reading outcomes, over and above the contribution of speech and language measures: it is therefore plausible that some of these risks associated with family risk lie outside the domain of speech, phonology and language. These may be genetic or environmental, and lie beyond the scope of the current investigation.

The current study focuses on the literacy outcomes of children with SSD in the initial stages of reading instruction at age 5 and 3 years later at age 8. We tested the following hypotheses:

1. There will be an overall effect of early SSD status on literacy outcomes at both time‐points.
2. When SSD co‐occurs with language impairment, this will result in poorer literacy outcomes than isolated SSD.
3. The presence of a family risk of dyslexia will contribute to the likelihood of a poor literacy outcome, over and above the contribution of speech and language status.
4. Within the group with SSD, there will be a worse literacy outcome if the initial SSD is more severe, if it persists to age 5, and if the speech errors can be characterised as ‘disordered’ rather than ‘delayed’.

## Methods

### Participants

Families were recruited to the study via advertisements, and through speech and language therapy services. A total of 245 children were recruited and tested 6 times at roughly annual intervals, between the ages of 3½ and 9 (an additional 15 children were recruited into the overarching study at Time 2, but are not included in the current analyses, because the focus is on SSD status at Time 1/age 3½). None met exclusionary criteria (MZ twinning, chronic illness, deafness, English as a second language, foster care or living in a children's home, or a known neurological or psychiatric disorder). Ethics approval for the study was provided by the ethics committee of the Department of Psychology at the University of York, and the NHS Research Ethics Committee. Parents provided informed consent for their child to be involved.

Following recruitment, families were classified as at family risk (FR) or not and each child was assessed for language impairment (LI). This led to an initial classification of children into four groups [FR (family risk only), LI (language impairment only), FRLI (family risk and language impairment) and TD control (typically developing); see Nash, Hulme, Gooch, & Snowling, 2013 for further details]. Although children were not originally recruited on the basis of speech sound disorder, they were assessed for this at T1, and those who met SSD criteria are the focus of the current study. It is important to note that many children fulfilled criteria for more than one risk category (family risk; LI, SSD; see Table 1 for details of participants). The study examines the progress of the sample at three time‐points of the overarching longitudinal study: T1 (age 3½), T3 (age 5½) and T5 (age 8) on a subset of measures. There was a small amount of missing data.

**Table 1:** Sample Descriptives. The SSD group is shown as an aggregated group, and subgroups according to co‐occurring LI and FR status

	Control (*N* = 68)	Total SSD group (*N* = 68)	SSD only (*N* = 13)	SSD + FR (*N* = 16)	SSD + LI (*N* = 22)	SSD + LI + FR (*N* = 17)
Age (years; months)	3;9 (3.23)	3;8 (3.13)	3;9 (3.72)	3;8 (2.58)	3;7 (2.13)	3;9 (4.05)
Gender (% male)	48	63	62	56	77	53
SES (postcode% rank^a^)	69.95 (28.31)	58.45 (29.25)*	75.54 (20.24)	57.63 (29.28)	59.12 (30.43)	45.29 (28.88)
Performance IQ^b^	115.57 (14.62)	101.27 (12.48)***	110.38 (8.27)	98.12 (12.34)	97.00 (13.90)	102.29 (10.37)
PCC at T1	90.49 (5.75)	52.43 (16.39)***	56.82 (13.73)	57.60 (15.43)	49.24 (18.39)	48.33 (15.56)
% of children in group with speech disordered errors at T1	n/a	27	31	19	23	29
% of children with SSD persisting to T3^c^	n/a	56	54	43	68	54

*p < .05; **p < .01; ***p < .001. ^a^SES based on postcode in United Kingdom, relative rank according to deprivation value; Lower = more deprived. ^b^Performance IQ is mean standard score, two WPPSI‐III Performance IQ subtests. ^c^Missing DEAP data at T3: *N* = 2 children (SSD + FR group), *N* = 3 (SSD + LI), *N* = 5 (SSD + LI + FR).

### Defining family risk of dyslexia, language impairment and speech sound disorder

#### Family risk status

Family risk status of dyslexia was ascertained on the basis of a first‐degree relative (biological parent or full sibling), as is standard practice in the field. Previous studies have mostly used parental self‐report measures to determine whether a first‐degree relative has dyslexia. We considered it appropriate to validate this process with objective assessment when possible because it is not uncommon for parents with a history of reading difficulties to be unaware that they have dyslexia. Thus, each parent who consented, regardless of whether or not he or she self‐reported as dyslexic, was assessed to ascertain family risk status (see below). Participating children were classified as at family risk: if a parent self‐reported as dyslexic, on the basis of the Adult Reading Questionnaire (Snowling, Dawes, Nash, & Hulme, 2012); for parents who consented to objective testing, a literacy composite score below 90 derived from two tests of nonword reading and spelling (Test of Word Reading Efficiency: TOWRE, Torgesen, Wagner, & Rashotte, 1999; and the spelling subtest from the Wide Range Achievement Test; WRAT‐4, Wilkinson & Roberston, 2006); and a parent with a literacy composite score below 96 AND a 1.5 *SD* discrepancy between literacy and nonverbal ability (Wechsler Abbreviated Scale of Intelligence: WASI, Wechsler, 1999); or a sibling with a diagnosis of dyslexia from an educational psychologist or specialist teacher.

#### Language impairment

LI status at age 3½ was determined on the basis of scoring below a cut‐off on two of the following four measures: the Expressive Vocabulary, Basic Concepts and Sentence Structure subtests of the Clinical Evaluation of Language Fundamentals – Preschool 2 UK (Wiig, Secord, & Semel, 2006), and the screener from the Test of Early Grammatical Impairment (TEGI; Rice & Wexler, 2001). The cut‐offs were a scaled score below 7 on the CELF subtests, or failure of the TEGI screener. In a subset of cases (*N* = 22) with insufficient data from these diagnostic tests, we used a combination of further language subtests to come to a clinical judgement of LI (Nash et al., 2013). Of the children with LI, six had low nonverbal ability and three were unable to complete the tests, while the remaining children in the LI group had nonverbal ability in the normal range (>80 on the Wechsler Preschool Primary Scale of Intelligence‐III, Wechsler, 2003).

#### Speech sound disorder

SSD status at age 3½ was based on percent consonants correct (PCC) scored on the screener and Articulation measure of the Diagnostic Evaluation of Articulation and Phonology (DEAP; Dodd, Hua, Crosbie, Holm, & Ozanne, 2002). Children were asked to name a series of pictures including all the consonants in British English; they were classified as having a SSD if they scored 2 standard deviations below the mean of the TD control group (PCC score < 74.32%). Sixty‐eight children fulfilled this criterion at T1.

At age 5½, 59 of these 68 children with a history of SSD were available and completed the DEAP screener and articulation measure for a second time. Children who had scored in the average range on the DEAP at Time 1 were not asked to complete the task again. The cut point for a designation of SSD at Time 3 was therefore based on having a PCC more than 2 standard deviations below the age‐appropriate mean in the DEAP manual, which equated to a score of 85% PCC. Thirty‐three children met this criterion for SSD at Time 3.

#### Differentiating speech delay and speech disorder

The DEAP Screener and Articulation tasks were used to provide a profile of each child's speech error pattern at Time 1. Error patterns were assigned if a child showed five examples of a particular error type (e.g. fronting, gliding or cluster reduction), excluding errors that could be explained by difficulties in articulating a sound in isolation. Error patterns were next classified as to whether they were age‐appropriate, developmentally delayed or disordered, according to the DEAP guidelines; five instances of a disordered error type led to a classification of ‘disordered speech’ (Dodd, personal communication, 2006). A child's speech profile was described according to their most serious error pattern (e.g. a child with both delayed and disordered error patterns would be classified as having disordered speech). About 60/68 children in the SSD group could be clearly classified as showing either delay or disorder at age 3½. Forty‐four children showed delayed and 16 showed disordered speech errors; two did not meet criteria for any speech pattern, one had an articulation disorder and four had incomplete speech data.

### Measures

#### Literacy outcome measures

At age 5½, children completed the following measures tapping emergent literacy skills, as part of a broader language and literacy assessment. Analyses were based on *z*‐scores standardised on the control group means; composites were created by averaging the *z*‐scores of the relevant measures.

*Phoneme awareness*: (a) Phoneme isolation – children had to identify the first (eight items) or last sound (eight items) of a given nonword (e.g. first sound of/guf/); testing was discontinued after four incorrect responses (α = .88) (b) Phoneme deletion, from the York Assessment of Reading Comprehension (YARC, Snowling et al., 2009) – children repeated a word, and then said it again leaving out a specified phoneme (e.g. ‘sheep’ ‘without the/p/’) (12 items) (α = .93).

*Word‐level reading*: (a) Early Word Reading from the YARC – children read aloud 30 single words, graded in difficulty, half of which were phonemically decodable and half irregular. Testing was discontinued after 10 consecutive errors (test–retest *r* = .95). (b) Single Word Reading Test (Foster, 2007) – children read 60 words of increasing difficulty; testing was discontinued after five consecutive errors/refusals (α = .98).

*Spelling*: In this bespoke task, children spelt five words, each represented by a picture (dog, cup, tent, book, heart); the score was the total number of words spelt correctly (α = .79).

At age 8½, children completed the following measures, as part of a broader assessment.

*Word‐level reading*: (a) Sight‐Word and Decoding subtests of the Test of Word Reading Efficiency (TOWRE; Torgesen et al., 1999) – children read as many words/nonwords from a list as they could in 45 s (α = .95). (b) Accuracy from the Passage Reading subtest of the YARC – children read aloud a series of short texts, with reading errors corrected up to a given number as specified in the manual. Reading accuracy was based on the two most difficult passages read (α = .87).

*Reading comprehension*: Passage Reading from the YARC – see above; passages which were read accurately were followed by a set of eight spoken comprehension questions. Comprehension ability scores were calculated based on the two most difficult passages read (α = .64).

*Spelling*: Spelling words subtest of the Wechsler Individual Ability Test (WIAT II; Wechsler, 2005) – children spelt words increasing in difficulty. Testing was discontinued after 10 consecutive errors. (Test–retest *r* = .93).

#### Socioeconomic status

Several measures of socioeconomic circumstances were collected in the present study. These included the father's and mother's occupation and educational qualifications as well as an index of deprivation associated with UK postcodes (Department of Communities and Local Government, Indices of Multiple Deprivation, 2007). Here we report socioeconomic status (SES) based on postcode as a centile score.

## Results

### Analysis plan

The first set of analyses characterise the group of children who met criteria for SSD at the age of 3½, in terms of their demographic characteristics, language and family risk status, speech profile and group‐level literacy outcomes at the ages of 5½ and 8. The second set of analyses take an individual differences approach, and use regression models based on the entire sample to examine SSD, language impairment and family risk status as predictors of continuous literacy outcomes. The final set of analyses use regression models based on the SSD group only, to examine the effect of the nature, persistence and severity of SSD on literacy outcomes. We report effect sizes where appropriate: d = 0.8 is considered large; 0.5 medium and 0.2 small.

### Characterising the SSD group

Descriptive statistics for the SSD and control groups are presented in Table 1 (the control group is defined as having no family risk of dyslexia, and not meeting criteria for LI or SSD at T1). The SSD and control groups were equivalent in age, but the control group was higher in SES and performance IQ, and had fewer boys (nonsignificant difference). Approximately half of the SSD group met criteria for language impairment at T1, and half had a family risk of dyslexia (with 22% of the SSD group meeting both LI and FR criteria). Of the SSD group, 73% had speech errors which indicated a speech delay and 27% had errors indicating speech disorder. The speech difficulties for over half the children persisted to the age of school entry, with 56% of the SSD group at 3½ also meeting criteria for SSD at age 5.

### Literacy outcomes of the SSD group

**Figure 1:**
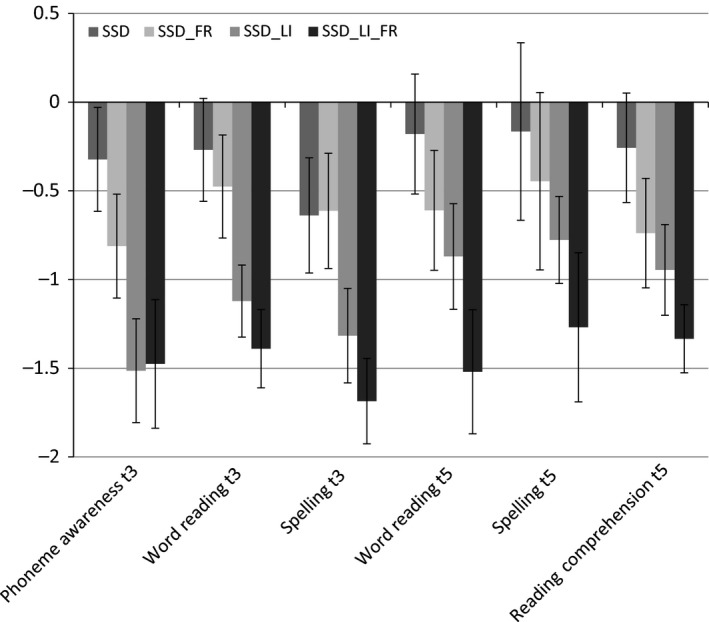
Mean scores for literacy outcome measures at age 8 for SSD according to subgroup. Scores are *z*‐scores, referenced to the TD group mean, with 95% CIs

The literacy skills of the SSD group were poorer than those of the TD group, in terms of phoneme awareness, word‐level reading and spelling at age 5, and word‐level reading, spelling and reading comprehension at age 8. Across measures, the SSD group was performing nearly 1 standard deviation below the TD group mean, a significant difference for all measures (Table 2). However, a key question is whether it is speech difficulties that compromise literacy development or whether the risk is actually carried by being at family risk of dyslexia or by broader oral language difficulties (both of which co‐occur with speech difficulties). The mean *z*‐scores on the literacy measures for subgroups of children with SSD classified according to co‐occurring language impairment and/or family risk status are presented in Figure 1. As can be seen from Figure 1, children with isolated SSD were performing consistently below the level of the typically developing group (−.2 to −.6 *SD*). Furthermore, the outcomes for children with SSD and co‐occurring LI were substantially lower, particularly at age 5, (−1 to −1.5 *SD*) and children who in addition to SSD and LI also had a family risk of dyslexia had the most severe and persistent literacy difficulties, with mean scores approximately −1.5 *SD* at both ages 5½ and 8. Although the effect sizes were generally moderate to large, most of the group comparisons illustrated in Figure 1 did not reach statistical significance, as indicated by the overlapping 95% confidence intervals.

**Table 2:** Literacy outcomes for the whole SSD group (*z*‐scores standardised relative to the control group mean)

	Mean (min–max)	*SD*	*t*	*df*	*p*
Phoneme Awareness T3	−1.08 (−3.73 to 1.27)	1.28	5.60^a^	102.50	.000
Word Reading T3	−0.84 (−2.26 to 1.43)	0.93	4.98	129	.000
Spelling T3	−1.08 (−2.10 to 2.03)	1.01	6.14	129	.000
Word Reading T5	−0.80 (−4.30 to 1.79)	1.27	4.09	124	.000
Spelling T5	−0.66 (−4.65 to 2.96)	1.38	3.13	125	.002
Reading Comprehension T5	−0.82 (−3.29 to 0.83)	0.95	4.58	119	.000

^a^Equal variances not assumed.

### Predicting literacy outcomes on the basis of SSD and LI status, and family risk of dyslexia

A series of simultaneous multiple regression models examined the influence of SSD and LI status at age 3½, and family risk of dyslexia (represented by dummy codes) on short‐term (age 5½) and long‐term (age 8) literacy outcomes (Table 3). These models explained between 8% and 20% of the variance in literacy scores. The use of simultaneous entry in these models means that the unique *R* ^2^ values represent the variance accounted for by each predictor after the effects of other predictors in the model have been controlled.

**Table 3:** Linear regression models predicting word‐level reading accuracy and spelling at T3 and T5, and reading comprehension at T5, *in the whole sample*: prediction from SSD and LI status at T1 and family risk of dyslexia

	Phoneme Awareness T3 (*N* = 224)		Word Reading T3 (*N* = 222)		Spelling T3 (*N* = 224)		Word Reading T5 (*N* = 218)		Spelling T5 (*N* = 219)		Reading Comprehension T5 (*N* = 212)	
	Stand. beta	Total *R* ^2^ = 11.3%	Stand. beta	Total *R* ^2^ = 16.7%	Stand. beta	Total *R* ^2^ = 20.1%	Stand. beta	Total *R* ^2^ = 10.1%	Stand. beta	Total *R* ^2^ = 8.4%	Stand. beta	Total *R* ^2^ = 17.3%
		UniqueR^2^		UniqueR^2^		UniqueR^2^		UniqueR^2^		UniqueR^2^		UniqueR^2^
SSD at T1	−.26***	5.8%	−.11	0.9%	−.20**	3.4%	−.15*	1.9%	−.07	0.4%	−.10	0.8%
LI at T1	−.24***	4.9%	−.31***	8.5%	−.32***	8.8%	−.16*	2.2%	−.20**	3.4%	.34***	10.3%
Family risk	−.09	0.9%	−.18**	3.3%	−.11	1.1%	−.20**	3.8%	−.18**	3.2%	−.16*	2.5%

*p < .05; **p < .01; ***p < .001.

The presence of SSD at T1 was a fairly weak predictor of outcomes, though it did account for statistically significant unique variance in phoneme awareness (5.8%) and spelling at age 5½, (3.4%) and word reading at age 8 (1.9%). In contrast, language impairment at T1 was a stronger predictor, accounting for statistically significant unique variance (between 2% and 10%) in all outcome measures. Family risk status had effects that were stronger than the effects of SSD but weaker than the effects of language impairment, accounting for statistically significant unique variance (between 0.9% and 3.8%) in word reading at age 5½ and word reading, spelling and reading comprehension at age 8.

These models were rerun including Performance IQ at T1 as a covariate. The pattern of significant predictive relationships remained unchanged, except for the fact that the relationship between language impairment at T1 and spelling at age 8 was reduced to nonsignificant levels.

### The nature, persistence and severity of SSD in relation to literacy outcomes

To assess whether the severity or type of speech difficulty (disordered vs. delayed) at age 3½, or its persistence to age 5½ (persistent vs. resolved) predicted subsequent literacy outcomes, we computed correlations between these variables and literacy outcomes among children with SSD only (Table 4). Severity of SSD at age 3½ did not correlate appreciably with any of the literacy outcome measures. Point biserial correlations showed that the persistence of SSD to age 5½ correlated significantly with phoneme awareness and word reading measured concurrently (16% and 21% of the variance respectively). In addition, disordered speech errors are associated with poorer word reading at age 5½ (7.6% of the variance). None of the predictors were significantly associated with literacy outcomes at age 8.

**Table 4:** Point biserial (nature and persistence of SSD) and Pearson (severity of SSD) correlations with measures literacy skills *in the SSD sample only*

	Severity	Disordered speech errors	Persistence
Phoneme Awareness T3	.09	−.06	−.40*
Word Reading T3	−.02	−.28*	−.45*
Spelling T3	.10	−.18	−.26 (*p* = .051)
Word Reading T5	.01	−.17	−.18
Spelling T5	.04	−.15	−.24
Reading Comprehension T5	.16	−.27 (*p* = .059)	−.01

* indicates *p* < .05.

## Discussion

We followed the progress of children with SSD through the early stages of literacy development from age 5½ to 8 years, comparing them with typically developing controls. Of particular interest was the outcome of children with isolated SSD compared with those of children who had co‐occurring language impairment (LI) and/or those at family risk of dyslexia.

### What is the effect of early SSD status on literacy outcomes?

We found that, as a group, children identified with SSD at the age of 3½ performed more poorly than typically developing peers on measures of phoneme awareness, word‐level reading and spelling around the point of school entry and on word‐level reading, spelling, and reading comprehension 3 years later, with large effect sizes. However, when the group was subdivided according to co‐occurring conditions, those with isolated SSD showed problems only at the younger age with phoneme awareness and spelling. Consistent with this, SSD status at age 3½ accounted for up to 5.8% of the variance in early phonemic and spelling skills when LI and FR status were controlled but only up to 1.9% of the variance at age 8 (though its effects on word reading were significant at *p* < .05). It follows that the risk of poor literacy in children with isolated SSD is low, with only modest and mostly short‐lived effects on literacy development, with the skills most closely related to speech in the early stage of development being affected, namely phoneme awareness and spelling. It seems that most children recover from this early set back.

### Does comorbid LI lead to poorer literacy outcomes than SSD alone?

In line with predictions, the outcome for children with SSD and co‐occurring language impairment was poorer than for SSD alone. The literacy skills of children with SSD and LI were significantly impaired relative to controls and children who in addition were at family risk of dyslexia showed the most significant impairments. When both SSD and FR dyslexia were controlled, LI status at age 3½ T1 accounted for significant amounts of variance in early literacy skills and continued to predict later development especially of reading comprehension.

### Does family risk of dyslexia contribute to the likelihood of a poor literacy outcome, over and above speech and language status?

Children with SSD who were also at family risk of dyslexia showed significant impairments relative to TD controls in phoneme awareness, reading and spelling skills which persisted except for spelling at age 8. The impairments were more marked if LI was also present. In and of itself FR status accounted for a small but significant amount of variance in literacy outcomes when speech and language difficulties were controlled, supporting the findings of Carroll et al. (2014) and Puolakanaho et al. (2007). It should be noted, however, that it does not predict either phoneme awareness or spelling at age 5½, plausibly because at this stage most variance in these skills is associated with speech status 2 years earlier when many children at family risk of dyslexia show speech difficulties.

### Does the initial severity or nature of the SSD or its persistence to age 5 predict literacy outcomes?

Contrary to prediction, the severity of SSD had a negligible effect on the development of literacy skills within the SSD group. The propensity to make disordered speech errors was a significant predictor of word reading – but not phoneme awareness – at age 5½, contrary to previous findings (Holm et al., 2008; Leitão et al., 1997). Persistence on the other hand predicted both phoneme awareness and word reading at age 5½, but not at age 8. It may be that the effects are short‐lived because children are able to draw on compensatory mechanisms, for example in orthographic learning, to aid literacy development despite early deficits (e.g. Peterson et al., 2009; Treiman, Pennington, Shriberg, & Boada, 2008).

Together, our findings are broadly consistent with those reported in previous studies of speech sound disorder (Bird et al., 1995; Bishop & Adams, 1990; DeThorne et al., 2006; Lewis et al., 2000; Nathan et al., 2004). However, it is clear that the impact of speech sound disorder on literacy development is relatively short‐lived (cf. Leitao & Fletcher, 2004) and we found no strong evidence that disordered (rather than immature) speech errors are indicative of more serious deficits in phonological awareness and literacy (cf. Holm et al., 2008; Leitao & Fletcher, 2004; Leitão et al., 1997; Preston et al., 2013). The situation is different when SSD co‐occurs with language impairment as it frequently does: language impairment carries a strong risk of literacy difficulties affecting both word reading and reading comprehension skills. Similarly, when SSD persists to school entry when reading instruction begins, it has concurrent effects on phoneme awareness and reading skills, consistent with the critical age hypothesis (Bishop & Adams, 1990).

The present study has limitations. First, although the sample of children with SSD was reasonable in size, the number of children in the different subgroups was small, limiting statistical power to detect the differences between these subgroups (e.g. between children with SSD and LI and children with SSD and family risk of dyslexia). Second, the study takes account of the persistence of SSD over time but only considers the impact of LI as ascertained at the first time‐point and not according to persistence (see Snowling, Duff, Nash, & Hulme, 2015 for discussion of the persistence of LI in this sample). Nevertheless, the findings add to the growing body of evidence that speech difficulties in preschool confer only a slight risk of poor literacy outcomes unless they are accompanied by language difficulties (Pennington & Bishop, 2009).

Together, our results indicate a relatively minor role for early SSD as a risk factor for the development of dyslexia, within a framework which suggests that risk factors accumulate to have a negative effect on literacy development (the multiple‐risk model). The impact of SSD per se appears to be primarily on early literacy‐related skills, with its effects lessening over time; in contrast, the impact of LI with which it is frequently comorbid increases over time and affects the development of both word reading and reading comprehension skills (Hulme et al., 2015). Over and above the effects of speech and language difficulties on literacy development, being at family risk of dyslexia is an additional risk factor for literacy difficulties. While its unique effects are small, it is important to consider what might account for this influence. One plausible mechanism is that family risk is associated with environmental influences that affect learning to read; indeed using data from the current sample, Dilnot, Hamilton, Maughan, and Snowling (2016) showed that once socioeconomic and home literacy environmental influences are accounted for, family risk of dyslexia no longer accounts for significant variance in reading readiness. Alternatively, the influence of family risk might be attributable to sources of variance, for example in executive and motor skills, not controlled in the present analyses.

## Conclusions

Speech difficulties impede communication and are a cause of concern for parents often leading to referral to speech and language services. We have shown that these difficulties have weak associations with the development of phonological skills in the early years and hence literacy can get off to a slow start. However, unless there are co‐occurring problems, children with speech sound disorder are not at high risk of reading difficulties. A family history of literacy problems (not unlikely given the genetic overlap between SSD and dyslexia) should alert the clinician to the need to monitor the development of a child with SSD through the early stages of literacy development. More importantly, a co‐occurring language impairment will place the child at high risk of difficulties learning to read and appropriate interventions should be put in place.

**Key points**

- Speech difficulties which persist to the point of school entry are associated with weak emergent literacy skills at the age of 5½, but these effects are no longer apparent at 8 years.
- Disordered speech errors are associated with poorer word reading at age 5½, but these effects are also short‐lived and not apparent at age 8.
- Early language impairment which co‐occurs with speech difficulties is predictive of poor word‐level literacy at both 5½ and 8, and of reading comprehension at age 8.
- Having a family risk of dyslexia has a small but significant effect on literacy at both ages, above and beyond the effects of speech and language.

## Supporting information


**Appendix S1**. Participant groups at Time 1 of the Wellcome Language & Reading Project sample overall (above), showing how they map onto the SSD groups used for analyses in this paper (below).
