# Supplementary material for: When does speech sound disorder matter for literacy? The role of disordered speech errors, co‐occurring language impairment and family risk of dyslexia
Source: J Child Psychol Psychiatry. 2016 Nov 7;58(2):197–205. doi: 10.1111/jcpp.12648 (PMC5297982; doi:10.1111/jcpp.12648)
Supplement: Supplementary file 1 — Appendix S1. Participant groups at Time 1 of the Wellcome Language & Reading Project sample overall (above), showing how they map onto the SSD groups used for analyses in this paper (below). [file JCPP-58-197-s001.docx]

**Appendix S1**. Participant groups at Time 1 of the Wellcome Language & Reading Project sample overall (above), showing how they map onto the SSD groups used for analyses in this paper (below).

**FRLI**

**N = 37**

**LI**

**N = 36**

**FR**

**N = 86**

**Not FR/LI**

**N = 86**

T1 data

N = 245^3^

*Main classifications used in overall Wellcome study, based on LI and FR status (see Nash et al., 2013).*

**SSD-FRLI**

**N = 17^2^**

**FRLI**

**N = 19**

**SSD-LI**

**N = 22^2^**

**LI**

**N = 13**

**SSD-FR**

**N = 16**

**FR**

**N = 70**

**SSD^1^**

**N = 13**

**Control**

**N = 73**

*Classifications used in current study, based on SSD status, as well as LI and FR status.*

*Notes*

1. In Nash et al., (2013), 10 of these SSD children were excluded because they did not fulfill criteria for LI or FR. The other 3 were referred as TD and included in the TD group.
2. One participant in each of these groups was missing DEAP data at T1: FR and LI status could be determined, but not SSD status.
3. 245 chidlren were recruited at Time 1 of the overall Wellcome Lnaguage & Reading Project, at the age of 3½. An additional 15 children were recruited at Time 2, bringing the total sample size to 260. However, these children were not included in the current paper, because the focus was on SSD and LI status at the age of 3½ (Time 1) .
